# The multifaceted potential of fenugreek seeds: From health benefits to food and nanotechnology applications

**DOI:** 10.1002/fsn3.3959

**Published:** 2024-01-10

**Authors:** Zargham Faisal, Rushba Irfan, Noor Akram, Hafiz Muhammad Irfan Manzoor, Mohib Ali Aabdi, Muhammad Junaid Anwar, Sharjeel Khawar, Asifa Saif, Yasir Abbas Shah, Muhammad Afzaal, Derese Tamiru Desta

**Affiliations:** ^1^ Department of Human Nutrition, Faculty of Food Science and Nutrition Bahauddin Zakariya University Multan Pakistan; ^2^ Faculty of Food Nutrition and Home Sciences University of Agriculture Faisalabad Pakistan; ^3^ Department of Food and Nutrition Government College University Faisalabad Pakistan; ^4^ Department of Agriculture and Food Technology Karakoram International University Gilgit‐Baltistan Pakistan; ^5^ Department of Food Science and Technology, Faculty of Food Science and Nutrition Bahauddin Zakariya University Multan Pakistan; ^6^ Natural and Medical Science Research Centre University of Nizwa Nizwa Oman; ^7^ Department of Food Science Government College University Faisalabad Pakistan; ^8^ School of Nutrition, Food Science and Technology Hawassa University Hawassa Ethiopia

**Keywords:** antioxidant, fenugreek seeds, health, nanotechnology, nutrition

## Abstract

The present lifestyle, dietary patterns, psychological pressure, environmental factors, and the widespread exploitation of processed substances in food production and farming have collectively contributed to a substantial expediting in the development of various health problems. Globally, researchers have been seeking natural pharmaceutical substances with the potential to be employed in treating lifestyle‐related diseases or delaying their onset. Fenugreek seeds have gained significant attention in various fields, including health, nutrition, and cutting‐edge nanotechnology applications, due to their versatile qualities. The current investigation offers a comprehensive discussion of the nutritional composition and therapeutic potential of fenugreek seeds, with an emphasis on their plentiful reservoir of bioactive compounds. This seed demonstrates promising medicinal potential in addressing a wide range of health issues. Significantly, these findings indicate noteworthy properties, such as antidiabetic, antioxidant, anti‐obesity, hypocholesterolemic, anticancer, and cardioprotective effects. Moreover, the components of fenugreek seeds are important in the development of a multitude of foods, which is the reason why they are used extensively in the area of food research. In addition to their nutritional value, their exploration of nanotechnology reveals a promising domain, utilizing the distinctive characteristics of seeds for many purposes, such as nanoparticle synthesis and oil for edible films and nanoemulsions. This review article focuses on a comprehensive analysis of fenugreek seeds, examining their wide‐ranging applications in the fields of health, nutrition, food, and nanotechnology.

## Graphical abstract

Fenugreek seeds have gained significant attention in various fields, including health, nutrition, and cutting‐edge nanotechnology applications. This review article focuses on a comprehensive analysis of fenugreek seeds, examining their wide‐ranging applications in the fields of health, nutrition, food, and nanotechnology.

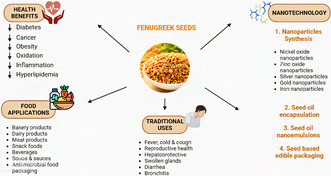

## 1 INTRODUCTION

Fenugreek (*Trigonella foenum‐graecum*), an ancient medicinal plant with long‐standing historical recognition, has earned the label of being an “old world” crop in the context of the “new world.” Fenugreek seeds have been utilized for their medicinal properties for centuries in various cultures (Ruwali et al., 2022). The small, golden‐brown seeds possess a diverse array of health benefits because of their abundant nutritional profile and the presence of bioactive compounds (Zandi et al., 2017). The nontoxic alkaloids and oil components present in the seeds contribute to the bitter taste. However, defatted seeds do not possess a bitter taste (Singh, Chamoli, et al., 2022; Singh, Yadav, et al., 2022). The United States Food and Drug Administration (FDA) has approved and generally acknowledged the safety of fenugreek seeds, extracts, oleoresins, and oils (Mandal & DebMandal, 2016).

The nutritional composition of fenugreek seeds has been studied in various works, in which the moisture content has been observed as 7.5%–10.6%, protein content as 0.78%–1.5%, fat content as 0.5%–1.6%, ash content as 0.5%–1.5%, and galactomannan as 73.6% (Rashid et al., 2018; Roberts et al., 2014).

Fenugreek gum typically contains galactomannans, with a typical galactose‐to‐mannose ratio of 1:1.1. Its molecular structure consists of a backbone made up of (14)‐β‐d‐mannan, with individual α‐d‐galactopyranosyl groups attached to the C‐6 position of the d‐mannopyranosyl residues (Dhull et al., 2022). Galactomannan has been proven to regulate blood sugar levels, by slowing down the absorption of carbohydrates in the digestive tract, leading to improved insulin sensitivity. Thus, making fenugreek seeds, particularly valuable for individuals with diabetes or those at risk of developing the condition (Luk et al., 2018).

Moreover, the soluble fiber in fenugreek seeds binds to cholesterol molecules in the gut, preventing their absorption into the bloodstream, and has been shown to lower total cholesterol and LDL (low‐density lipoprotein) cholesterol levels, reducing the risk of heart disease (Wang et al., 2023). Fenugreek seeds have been shown to hold appetite‐suppressing properties and can help control hunger and reduce food intake. Additionally, they may increase thermogenesis, promoting the burning of calories and aiding in weight management efforts (Al‐Snafi et al., 2022).

However, fenugreek seeds are rich in antioxidants, including polyphenols and flavonoids, which combat oxidative stress (OS) and reduce the damage caused by free radicals; therefore, the antioxidant activity (AOA) may help protect cells from aging and lower the risk of chronic diseases (Hozzein et al., 2020).

Some studies suggest that fenugreek seeds possess anti‐inflammatory properties. They may help reduce inflammation in various parts of the body, potentially benefiting conditions like arthritis and respiratory issues (Kaveh et al., 2022). Because the combined effects of blood sugar regulation, cholesterol management, and antioxidant activity contribute to fenugreek seeds’ potential to support heart health, they may, therefore, help reduce the risk of cardiovascular diseases (Hanafi et al., 2022). Fenugreek seeds may have a positive impact on hormonal balance, and studies have shown their potential to alleviate symptoms of menopause and polycystic ovary syndrome (PCOS) (Nannar et al., 2023).

Moreover, fenugreek gum finds widespread application in the food industry as a valuable additive for stabilizing and providing dietary fiber in various food products. Both producers and consumers favor it due to its cost‐effectiveness and natural properties. This versatile gum serves as an additive, functioning as a stabilizer, emulsifier, and hydrocolloid, effectively altering water behavior—a common component in diverse foods (Ağagündüz et al., 2023). In the realm of baking, incorporating fenugreek gum into biscuits or cake dough enhances its workability, facilitating easy release from molds and clean slicing postbaking. Furthermore, its high water‐binding capacity makes it a preferred stabilizer in frozen treats like ice cream (Banyal et al., 2022; Goksen et al., 2023; Sharanagat et al., 2022).

Fenugreek gum boasts excellent water retention capabilities in both hot and cold environments, making it a valuable lubricant, stabilizer, and binder in the preparation of items such as hotdogs, stuffed meats, and sausages (Frangopoulos, 2022). Hydrocolloids, like fenugreek gum, offer various functional benefits, including controlling syneresis and preventing fat migration during the storage of meat and meat products (Eghbaljoo et al., 2022). In the beverage industry, fenugreek gum serves as an effective tool for viscosity control and thickening, owing to its inherent properties. Additionally, it exhibits resistance to degradation even in low pH conditions commonly found in beverages (Singh, Chamoli, et al., 2022; Singh, Yadav, et al., 2022).

Intriguingly, as we delve into the world of fenugreek seeds, we will also explore their role in nanotechnology as reducing and capping agents for different nanoparticles and formation of nanoemulsions and edible films, thereby extending their impact beyond traditional medicine and culinary delights. The novelty of this review is that it scrutinizes the scientific evidence and delves into the wisdom that underscores the myriad benefits of fenugreek seeds and multidimensional applications. As we embark on this exploration, it becomes increasingly evident that fenugreek seeds are not merely culinary staples but represent a treasure trove of potential for human health and well‐being.

## 2 NUTRITIONAL COMPOSITION OF FENUGREEK SEEDS

A diverse range of nutrients and bioactive compounds in fenugreek are vital for improving the overall well‐being and efficacy of biological systems. Fenugreek seeds exhibit a protein content ranging from 22% to 26%, accompanied by a carbohydrate composition of 58%, a fiber content of 25%, and a lipid content of 0.9%. The fenugreek leaves exhibit carbohydrate, protein, and fiber contents of 6%, 4.4%, and 1.1%, respectively (Wani & Kumar, 2018).

### 2.1 Protein and amino acids

The protein quality of fenugreek seeds, which varies between 260.3 and 295.0 g/kg, is reliant on the protein fractions and amino acid compositions present. Based on a conducted study, it was shown that fenugreek seeds possess an amino acid profile consisting of albumins (438 g/kg), globulins (272 g/kg), glutelins (172 g/kg), and prolamins (74 g/kg). A comparative analysis revealed that the observed disparities in the functional attributes of wheat durum, fenugreek, and white lupine can be attributed to dissimilarities in the amino acid compositions of this food source (Kumari et al., 2022). Elkadousy et al. (2020) conducted an evaluation of protein quality in fenugreek and other legumes, including white lupine. Their findings indicate that white lupine had a greater protein content compared to fenugreek. The white lupine plant was seen to have 268.50 g/kg of essential amino acids, while fenugreek seeds had the highest amounts of both total and essential amino acids, measuring 576.00 and 304.80 g/kg, respectively. The amounts of cysteine and methionine in fenugreek were found to be around 49% and 128% higher, respectively, compared to white lupine. The presence of abundant free amino acids, particularly isoleucine, and histidine, in fenugreek may potentially stimulate the secretion of insulin (Żuk‐Gołaszewska & Wierzbowska, 2017).

Fenugreek seeds are commonly utilized as a nutritional supplement due to their high lysine content, which exhibits a comparable quality of lysine to that found in soybeans (Mandal & DebMandal, 2016). According to the findings of Feyzi et al. (2018), a particular strain of fenugreek protein was identified as a noteworthy protein source due to its remarkable functional attributes. This determination was made based on the significant presence of amino acids, such as arginine, threonine, glutamic acid, aspartic acid, and leucine, in fenugreek seeds. Fenugreek seeds can be utilized to enhance the nutritional composition of snack foods such as cakes, bread, and biscuits, as well as cereals. This is due to the seeds' high content of lysine, an essential amino acid while being relatively low in histidine and methionine. In comparison to unprocessed seeds, germinated fenugreek seeds exhibit a significantly elevated content of total protein (29%) and total lysine (64.80 g/kg of protein) when subjected to processing (Atlaw & Kumar, 2017).

### 2.2 Lipids and fatty acids

Fenugreek seeds contain lipids at a concentration of 100 g/kg. The predominant lipids in terms of composition are diglycerides, glycolipids, phospholipids, and triglycerides, accounting for around 6.3%, 5.4%, 10.5%, and 86.1%, respectively. Fenugreek seeds contain trace amounts of monoglycerides, free fatty acids, and sterols. In a separate investigation, the study revealed that the content of linoleic acid C18:2 ranged from 34.85% to 42.2%, while the content of α‐linolenic acid C18:3 ranged from 22.0% to 30.8%. Additionally, the content of myristic acid C14:0 was found to be between 0.10% and 1.38%, the content of palmitic acid C16:0 ranged from 3.85% to 13.10%, and the content of oleic acid C18:1 ranged from 13.30% to 19.0% (Bienkowski et al., 2017). In another study, the composition of fatty acids in fenugreek seeds consisted of oleic acid (C18:1) at a proportion of 35.1%, linoleic acid (C18:2) at 33.7%, α‐linolenic acid (C18:3) at 13.8%, palmitic acid (C16:0) at 9.6%, stearic acid (C18:0) at 4.9%, and arachidic acid (C20:0) at 2.0%. In a separate investigation, the proportion of linoleic acid was determined to be 34.85%, while the cumulative amount of unsaturated fatty acids was found to be 92.99% (Aljuhaimi et al., 2018).

### 2.3 Phytonutrients and fiber

**FIGURE 1:**
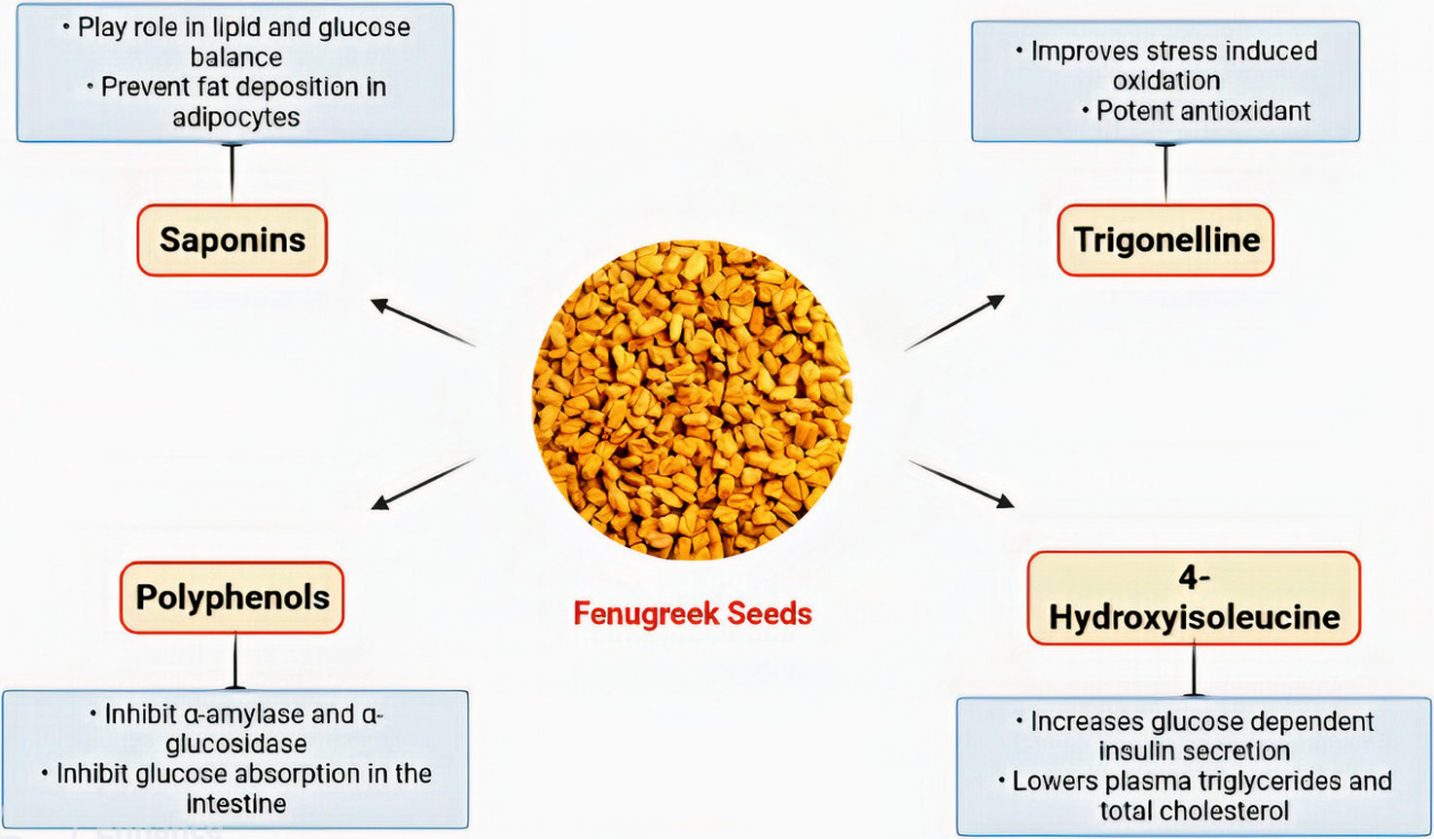
Bioactive compounds of fenugreek seeds and their health benefits.

Fenugreek seeds are known to be rich in phytonutrients that contribute to their potential health benefits (Mallik et al., 2020). Phytonutrients are naturally occurring compounds found in plants and have been shown to possess numerous health‐promoting properties. Bioactive compounds are present in fenugreek seeds and their health benefits are presented in Figure 1. One of the key phytonutrients present in fenugreek seeds is saponins (Yao et al., 2020). Saponins are known for their cholesterol‐lowering effects and are believed to inhibit the absorption of cholesterol in the intestines which may help reduce overall cholesterol levels in the body (Nagulapalli Venkata et al., 2017).

Fenugreek seeds also contain flavonoids that are powerful antioxidants. Antioxidants help neutralize harmful free radicals in the body, which can cause OS and damage to cells. Flavonoids have been associated with various health benefits, including anti‐inflammatory and anticancer properties. These antioxidant properties of fenugreek seeds may contribute to their potential role in supporting a healthy immune system and reducing the risk of chronic diseases (Akbari et al., 2019).

Fenugreek seeds are known to contain alkaloids, including trigonelline and choline. Trigonelline has been shown to have antidiabetic properties, as it may help regulate blood sugar levels by improving insulin sensitivity (Jagtap et al., 2022). Choline is an essential nutrient that plays a role in various physiological processes, including brain function, liver health, and metabolism (Wallace et al., 2018). Additionally, accounting for 50% of their composition, fenugreek seeds consist of dietary fiber. This dietary fiber is comprised of two components: insoluble fiber, making up 30%, and soluble fiber, making up 20%. The primary constituent of the soluble fiber is galactomannan. Galactomannan, a soluble fiber, comprises approximately 45%–60% of the fenugreek seed composition. Fiber is important for digestive health as it promotes regular bowel movements, helps regulate blood sugar levels, and may contribute to weight management (Korczak et al., 2017).

## 3 THERAPEUTIC POTENTIAL OF FENUGREEK SEEDS

**FIGURE 2:**
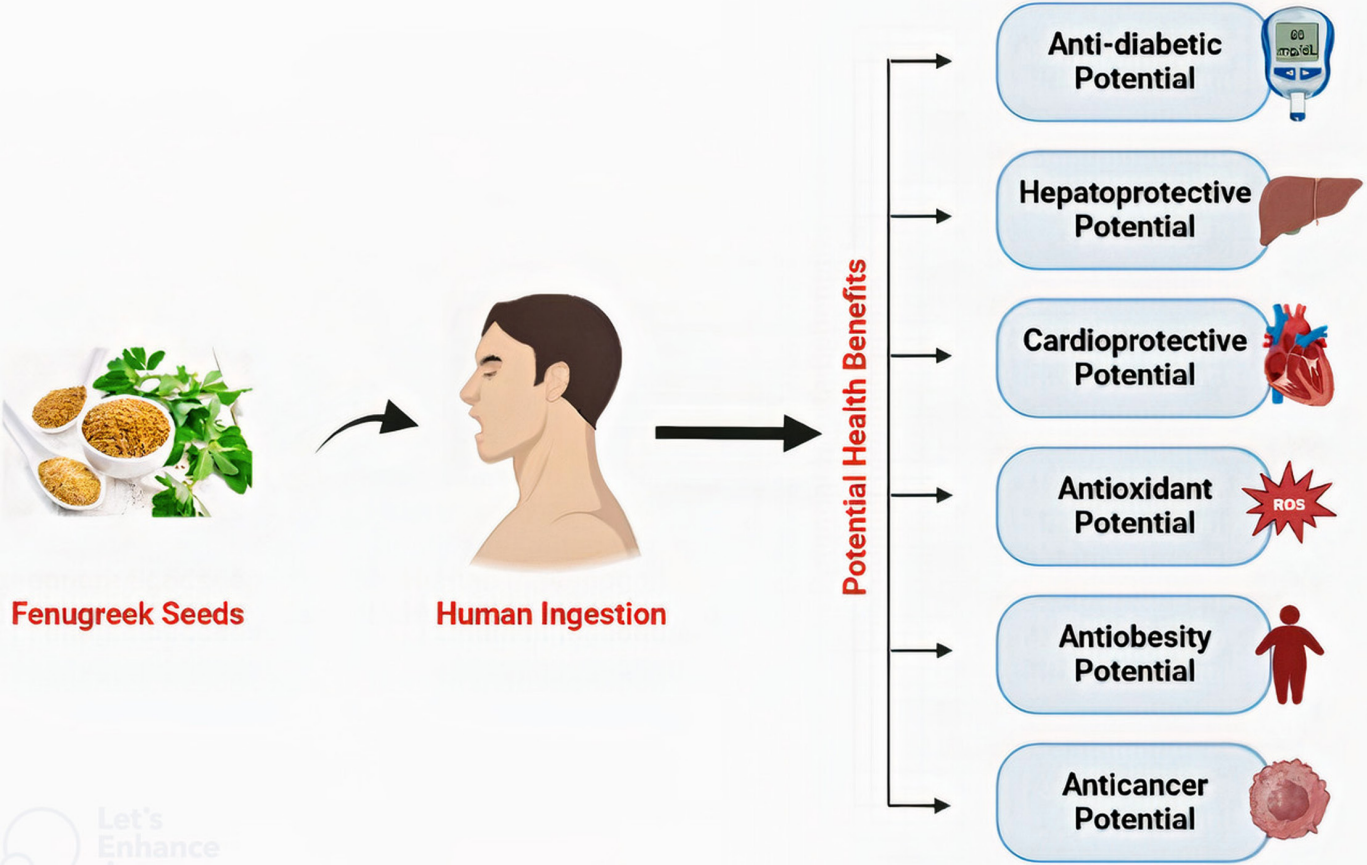
Reported health benefits associated with consumption of fenugreek seeds.

The therapeutic potential of fenugreek seeds depends on the bioactive components. These include proteins, amino acids, flavonoids, steroidal saponins, coumarin, lipids, vitamins, minerals, galactomannan fiber, and alkaloids, such as trigonelline. Significant pharmacological and clinical evidence has highlighted the medicinal application of fenugreek seeds as antidiabetic activity (Singh, Chamoli, et al., 2022; Singh, Yadav, et al., 2022), hypolipidemic activity, anti‐obesity activity, anticancer activity (Varadarajan et al., 2023), anti‐inflammatory activity, antioxidant activity, and antibacterial activity (Salam et al., 2023) due to its rich composition of phytochemicals. Figure 2 presents reported health benefits that have been linked with the consumption of fenugreek seeds. The fenugreek seeds were also traditionally applied for impotence, prolactin (Çayıroğlu et al., 2023), lowering blood sugar, and treatment of some symptoms like eczema (Mayathevar, 2022), gout (Ruwali et al., 2022), diarrhea, abdominal distension, swollen glands, bronchitis (Mandal et al., 2023), and stomach discomfort (Yao et al., 2020). Various clinical studies supporting the therapeutic potential of fenugreek seeds have been discussed in Table 1.

**TABLE 1:** Various clinical studies supporting the therapeutic potential of fenugreek seeds.

Supplement	Study type	Subject	Treatment	Outcomes	Reference
Fenugreek seed powder	Double‐blind, randomized controlled clinical trial (RCT)	60 patients‐mechanically ventilated	3 mg twice daily for 5 days	Reduces gastrointestinal (GI) bleeding, bloating, and heartburn	Zarghi et al. (2021)
Fenugreek seed extract	Randomized exploratory study	54 patients	500 mg/day for 12 weeks	Improves blood glucose metabolism	Pickering et al. (2022)
Gel from fenugreek seed	A double‐blind placebo‐controlled clinical trial	60 patients with trichoptilosis	500 μg/mL for 2 months	Herbal gel from fenugreek seed reduces the incidence of hair loss and trichoptilosis	Hajizadeh et al. (2022)
Fenugreek seed extract (metformin and the hydroalcoholic)	Mice study	52 diabetics induced Wistar rats	50 mg/kg, 100 mg/kg, and 200 mg/kg of the extract for 48 h	Reduces oxidative damage in brain and reduces memory loss resulting from diabetes	Bafadam et al. (2019)
Metformin‐fenugreek seed extract	Mice study	42 diabetics induced male rats	50 mg/kg, 100 mg/kg, and 200 mg/kg, 300 mg/kg of the extract for 6 weeks	Reduces oxidative stress, improves metabolism, and reduces apoptosis	Bafadam et al. (2021)
Fenugreek seed extract	Clinical trial	100 healthy males aged 45–80 years	600 mg/day for 12 weeks	No change observed in benign prostate hyperplasia	Rao and Grant (2020)
Fenugreek seed extract in capsule	A double‐blind, randomized placebo‐controlled clinical trial	58 oligo‐anovulatory polycystic ovary syndrome (PCOS) women	1 capsule for 8 weeks	PCOS improved in women	Bashtian et al. (2013)

### 3.1 Antidiabetic potential

Diabetes mellitus is recognized as a persistent metabolic illness characterized by elevated levels of blood glucose. This condition arises from impaired insulin function and/or secretion, occurring throughout periods of fasting and after meals (Faisal et al., 2022). Fenugreek has gained significant recognition for its utilization as an antidiabetic intervention for individuals afflicted with both type I and II diabetes. It has been widely employed as a reservoir of antidiabetic constituents, derived from its seeds, leaves, and extracts, across several experimental setups (Visuvanathan et al., 2022). In early 1986, it was found that ripe fenugreek, fenugreek gum, defatted fenugreek seeds, and fenugreek seeds exhibited the capacity to effectively lower blood sugar levels and enhance insulin response in individuals with diabetes (Sharma, 1986). Soluble fiber‐rich fenugreek seeds are understood to have an antidiabetic effect. Studies have revealed that the dietary fiber of fenugreek will delay gastric emptying and suppress the release of gastric inhibitory peptides and insulinotropic hormones (Srinivasan, 2019a, 2019b). Fenugreek seeds contain a large amount of soluble dietary fiber (galactomannan), diosgenin, trigonelline, 4‐hydroxyisoleucine, flavone C‐glycosides, and other ingredients that show hypoglycemic activity and improve the insulin response (Laila et al., 2022). Fenugreek seeds are found to possess a considerable amount of fiber, with a composition of 51.7%. This fiber content is further divided into two types: 19.2% mucilaginous fiber and 32.5% neutral fiber. According to reports, the utilization of a fenugreek seed decoction has demonstrated the potential for improved diabetes, reducing glycosuria in moderate cases, and mitigating the severity of diabetes (Srinivasan, 2019a, 2019b).

The soluble dietary fiber galactomannan, derived from fenugreek seeds, has demonstrated the ability to reduce blood glucose levels in individuals with diabetes through various mechanisms. These include the inhibition of digestive enzymes, the delay of carbohydrate emptying from the stomach, the fostering of regular bowel movements, and the regulation of intestinal flora (Yao et al., 2020). Fenugreek seeds possess diosgenin, a chemical that exhibits many physiological effects, such as the preservation of pancreatic islet beta cells, upregulation of hepatic glucose kinase, downregulation of hepatic glucose heteroplasia, and enhancement of anti‐oxidase activity. Diosgenin demonstrated efficacy in alleviating diabetic symptoms through its anti‐inflammatory properties and promotion of adipocyte differentiation (Gan et al., 2020).

Trigonelline has been shown to exhibit antidiabetic efficacy by augmenting the insulin signaling pathway, mitigating endoplasmic reticulum stress, and diminishing oxidative damage in individuals with type 2 diabetes. The alterations exerted an impact on the regenerative capacity of pancreatic islet beta cells, the release of insulin, and the activity of glucose‐metabolizing enzymes (Alam et al., 2022). Diosgenin possesses the capacity to safeguard pancreatic islet beta cells, enhance the activity of hepatic glucose kinase, reduce the activity of hepatic glucose heteroplasia, and augment the activity of anti‐oxidase (Fuller & Stephens, 2015). Flavone C‐glycosides possess the capacity to hinder the activity of digestive enzymes, stimulate insulin signaling, and reduce the synthesis of advanced glycation end products. Furthermore, it has been observed that 4‐hydroxyisoleucine induces the pancreas to secrete insulin. The essential oils derived from fenugreek seeds possess a high concentration of omega‐3 fatty acids, known for their capacity to inhibit the function of pancreatic alpha‐amylase and maltase in diabetic rats, while also protecting the pancreatic islet beta cells (Landazuri et al., 2021).

Due to heightened hypoglycemic and antihyperglycemic properties, the aqueous extract derived from fenugreek seeds exhibits potential as an adjunctive therapeutic agent for managing diabetes, potentially enabling a significant reduction in the dosage of conventional pharmaceutical interventions. Fenugreek seeds provide a substantial protein content, rendering them a viable alternative to pulses in the dietary regimens of those with diabetes. Individuals diagnosed with diabetes may see positive outcomes in the treatment of their illness with the implementation of a supportive therapeutic approach, which involves the incorporation of a daily dietary intake of 25–50 g of fenugreek seeds (Sundaram et al., 2020).

There exist empirical data supporting the notion that the ingestion of fenugreek seeds has the potential to reduce levels of cholesterol in the bloodstream, induce a decline in blood glucose levels, and enhance lipid metabolism. Moreover, previous studies have provided evidence to support the notion that the ingestion of fenugreek has the potential to reduce plasma and liver cholesterol levels (Salman & Qadeer, 2021). This reduction in cholesterol levels has been observed to enhance insulin sensitivity in rats afflicted with metabolic disorders. Fenugreek seeds have been observed to trigger notable enhancements in the insulin response caused by glucose and a decrease in glucose tolerance in both humans and animals. These findings suggest a potential hypoglycemic impact associated with the consumption of fenugreek seeds. Several clinical investigations conducted on human subjects have demonstrated the potential efficacy of fenugreek seeds in reducing total cholesterol levels in individuals with moderate atherosclerosis or either insulin‐dependent or noninsulin‐dependent diabetes. A human study has demonstrated the potential of processed fenugreek seeds, devoid of fat content, to elevate levels of high‐density lipoprotein (HDL) cholesterol, commonly referred to as “good” cholesterol (Singh et al., 2023).

Extensive evidence indicates that the hypoglycemic impact of fenugreek can be attributed to its high content of fiber and gum, constituting around 52% of the seeds. The consumption of fenugreek in one's diet has the potential to reduce blood sugar levels due to its ability to delay gastric emptying by directly impeding glucose absorption. Furthermore, the consumption of dietary fiber that undergoes gel formation has been found to impede the secretion of insulinotropic hormones and stomach‐inhibitory polypeptides within the human body. Additionally, there exists a hypothesis suggesting that the potential hypoglycemic effect could be attributed to the stimulation of insulin synthesis and/or secretion from the pancreatic beta cells of Langerhans, as well as the enhancement of tissue sensitivity to endogenous insulin. The efficacy of fenugreek seeds in managing type 1 diabetes has been demonstrated by their ability to inhibit alterations in the enzymatic activity associated with glycolysis, gluconeogenesis, and lipogenesis in the liver and kidneys. The association between this phenomenon and the elevated lignan levels in the seeds has been established (Srinivasan, 2019a, 2019b).

### 3.2 Antioxidant potential

Free radicals as oxidants and inhibitors of enzymes, due to the high reactivity, lead to biomolecular oxidation such as protein, lipids, DNA, and amino acids, which finally results in cell damage. Hence, it is vital to maintain a harmonious equilibrium between free radicals and antioxidants to ensure proper physiological functioning and safeguard the body against OS. The concept of “oxidative stress” relates to a condition characterized by an imbalance between the quantities of oxidants and antioxidants within the human body, potentially leading to various ailments (Liu et al., 2021). OS has a substantial role in the pathogenesis of diabetes, cancer, and the associated complications of these conditions. Fenugreek seed supplementation has been reported to possess antioxidant potential by downregulating glutathione reductase, and glutathione peroxidase, and by upregulating superoxide dismutase, vitamin C, and phenolic properties in the liver (Tewari et al., 2020). Fenugreek seeds possess a significant amount of phenolic and flavonoid compounds, which collectively enhance the spice's inherent antioxidant capacity (Akbari et al., 2019). There exists a hypothesis suggesting that fenugreek seeds possess a potent antioxidant property that exerts a favorable impact on the liver and pancreas. The association between antioxidant properties and the health advantages of natural products has prompted investigations into the antioxidant properties of germinated fenugreek seeds. These studies have revealed that germinated seeds offer greater benefits compared to dried seeds, primarily due to the enhanced bioavailability of various constituents of fenugreek. The research findings indicate that fenugreek seeds that have undergone germination exhibit a notable degree of antioxidant activity. This observed phenomenon is hypothesized to be linked to the presence of flavonoids and polyphenols within the seeds (Lohvina et al., 2021).

Research has demonstrated that the consumption of fenugreek seeds has the potential to mitigate excessive lipid peroxidation and modulate the levels of endogenous antioxidant molecules in the circulatory system. The soluble components found in fenugreek seeds are linked to their antioxidant effects. The administration of fenugreek to animals with diabetes resulted in the restoration of their modified antioxidant levels and activities of antioxidant enzymes. This observation implies that fenugreek seeds possess beneficial antioxidant properties that may be utilized in the management of diabetic conditions (Saadh, 2020). The experimental rats exhibited enhanced improvement in diabetic hyperglycemia and associated metabolic abnormalities through the consumption of dietary fenugreek, which was further enhanced by the addition of onion. It has been proposed that the mitigation of OS plays a role in the antidiabetic effects. Additionally, the nutraceutical impact of fenugreek on OS induced by diabetes was found to be more pronounced when combined with onion consumption (Srinivasan, 2019a, 2019b).

The cardioprotective effects of fenugreek seeds have been observed in rats with experimentally induced myocardial infarction. The administration of isoproterenol resulted in increased levels of lipid peroxides and decreased levels of antioxidant molecules, leading to myocardial necrosis. Additionally, the activities of antioxidant enzymes in both the serum and heart were altered. However, these detrimental effects were mitigated by the inclusion of fenugreek in the diet. The consumption of fenugreek seeds improved the diminished antioxidant levels, with a greater cardioprotective impact observed when fenugreek seeds were combined with garlic. The examination of fenugreek's potential in reducing LDL oxidation has been carried out in rats, as LDL oxidation is recognized as a significant contributor to the development of arteriosclerosis. The findings indicate that the consumption of fenugreek seeds protects against the oxidation of LDL cholesterol (Riaz et al., 2020).

In rats with colon cancer induced by 1,2‐dimethylhydrazine (DMH), the capacity of fenugreek seed to decrease hepatic OS and its antioxidant capabilities were investigated. The administration of DMH resulted in an upsurge in lipid peroxidation as well as a reduction in the amount of glutathione, catalytic activity, glutathione peroxidase, glutathione transferase, and sphincter of Oddi dysfunction in the liver of the animals. The fenugreek seed powder containing food has been shown to reduce the risk of developing a tumor in the colon and to prevent lipid peroxidation in rats that have been treated with DMH. In the rats' livers, this diet also increased the activities of catalase (CAT), glutathione peroxidase (GPx), and glutathione transferase (GST) (Syed et al., 2020).

### 3.3 Anti‐obesity potential

The dietary fibers found in fenugreek seeds, such as galactomannan, have been observed to possess notable anti‐obesity properties. This is attributed to their ability to create a gel‐like substance within the intestines, which subsequently hinders the absorption of glucose and lipids. The aqueous extract derived from fenugreek seeds demonstrated efficacy in mitigating fat accumulation caused by a high‐fat diet and ameliorating dyslipidemia in rats. These effects were associated with its ability to hamper fat digestion and absorption, enhance glucose and fat metabolism, augment insulin sensitivity, boost antioxidant capacity, and diminish lipase activity (Yao et al., 2020). A separate investigation was conducted to assess the suppressive impact of an aqueous seed extract on the accumulation of adipose tissue and the development of dyslipidemia in rats that were overweight due to a high‐fat diet. The intervention demonstrated a significant reduction in body weight increase, leptin levels, body mass index (BMI), lipid levels, serum insulin levels, and blood glucose levels (Riaz et al., 2020).

The study showed that INDUS810, a naturally occurring substance found in fenugreek seeds, effectively reduced the increase in weight caused by a high‐fat diet in various adipose tissues, including epididymal white adipose tissue, liver, and interscapular brown adipose tissue. Additionally, it was found to decrease serum levels of low‐density lipoprotein (LDL) cholesterol. The study observed that the protein levels of peroxisome proliferator‐activated receptor‐gamma (PPARγ), co‐activator 1 beta (PGC1), sirtuin 1 (SIRT1), and sirtuin 3 (SIRT3) were affected by INDUS810, resulting in the inhibition of lipid accumulation and the promotion of lipolytic activity in mature adipocytes. The mechanism of action for combating obesity is dependent on the activation of adenosine monophosphate‐activated protein kinase (AMPK) (Idris et al., 2021). Yamogenin decreases lipid buildup in the liver by regulating gene expression involved in fatty acid production in hepatocytes (Srinivasa & Naidu, 2021).

Further investigation has revealed that the presence of fiber in fenugreek significantly reduces appetite, particularly in experimental subjects with obesity. The efficacy of fenugreek dietary supplementation in promoting short‐term weight loss has been scientifically shown. Upon administering fenugreek powder to a group of obese rats over a period of 14 weeks, notable alterations in nutritional parameters, body dimensions, and a reduction in body mass were seen (Arya et al., 2023).

### 3.4 Hypocholesterolemic potential

The condition characterized by an atypical decrease in blood cholesterol levels is referred to as hypocholesterolemia. In a study conducted on mice, the oral administration of methanolic and aqueous extracts of seeds at a dosage of 1 g/kg of body weight demonstrated a hypoglycemic effect. Fenugreek seeds are known to possess a significant quantity of dietary fiber, with galactose and mannose being the primary constituents of the gum found in these seeds. These substances are linked to decreased levels of cholesterol in the body (Wani & Kumar, 2018). Fenugreek extract, which contains significant amounts of 4‐hydroxyisoleucine and trigonelline, has been shown to effectively block the oxidation of LDL in vitro (Das et al., 2018).

Fenugreek galactomannan is a heteropolysaccharide known for its ability to lower blood glucose levels and regulate surface activity within the small intestine. It has been found to yield significant health advantages, particularly in the lowering of LDL cholesterol levels in individuals with hypercholesterolemia. Additionally, galactomannan has been observed to positively impact blood lipids, blood pressure, and fibrinolysis (Li et al., 2023). Fenugreek seeds possess the ability to regulate elevated blood cholesterol levels through the presence of antioxidants. The hypocholesterolemic properties of flavonoids derived from ethyl acetate extracts of seeds have been observed (Yaldiz & Camlica, 2021).

Worldwide experts have undertaken profound studies on the effectiveness of fenugreek sprout extract in combating cholesterol. In vivo studies have been conducted, encompassing a range of animal species including rabbits, in addition to rats and mice. The incorporation of fenugreek seeds into the dietary regimen of the mice resulted in a significant reduction in cholesterol levels, with reductions of up to 42% and 58% observed in both the control group and the hypocholesterolemia group, respectively (Algridi & Azab, 2021). The detection of cholesterol in plasma serves as a reliable marker for the presence of coronary heart disease. The impact of fenugreek seed extract on the plasma lipid profile has been investigated by researchers. The administration of fenugreek seeds and their extracts resulted in a considerable reduction in plasma cholesterol, triglyceride, and LDL cholesterol levels (Rouag et al., 2020).

The hypocholesterolemic effect of various fractions of fenugreek seeds was examined, and it was shown that only the fiber and saponin components, after defatting, demonstrated cholesterol‐lowering activity. Therefore, it may be surmised that the cholesterol‐lowering effect is linked to the fiber and saponin components found in fenugreek seeds (Srinivasan, 2019a, 2019b). The study provided evidence of the hypocholesterolemic effect of dietary fenugreek seeds when consumed with a high‐cholesterol diet (HCD) at a concentration of 10% in Wistar rats. The use of fenugreek seeds in diet demonstrated considerable ability to mitigate the adverse effects of high‐cholesterol levels and increased hepatic cholesterol resulting from HCD. Furthermore, the effectiveness of this mitigation was enhanced when garlic was concurrently incorporated as an intervention. Dietary fenugreek was found to have a favorable effect on the high‐cholesterol levels in the heart. The inclusion of fenugreek seeds in the diet of rats on a high‐fat diet (HFD) was found to have a notable impact on reducing cholesterol levels (Reddy et al., 2019).

### 3.5 Anticancer potential

**FIGURE 3:**
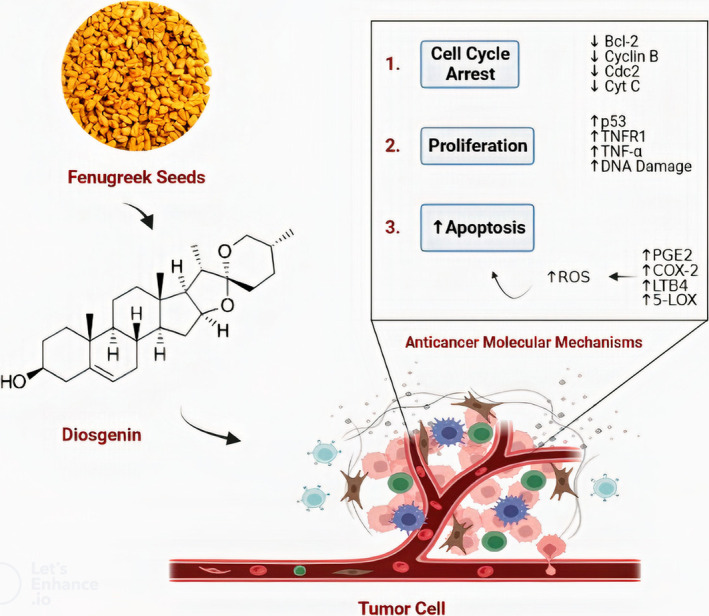
Anticancer activity of diosgenin through multiple molecular mechanisms.

The presence of phytoestrogens and saponins in fenugreek has been identified as the chemical constituents accountable for the herb's anticancer properties. Saponins exhibit a specific inhibitory effect on cell division in tumor cells and possess the capacity to induce apoptotic mechanisms, hence potentially triggering controlled cell death (Kannan et al., 2019). The anticancer activity of diosgenin, a steroidal saponin found in fenugreek seeds, is presented in Figure 3. Protodioscin, derived from fenugreek seeds, effectively inhibits the growth of HL60 cells by inducing apoptotic changes (Syed et al., 2020). Numerous investigations have been undertaken to examine the anticancer properties of chemical components found in fenugreek, yielding promising outcomes. For instance, studies have indicated that molecules belonging to the class of alkaloids, known as “trigonelline,” exhibit potential therapeutic applications in the field of cancer treatment (Khan et al., 2022).

Fenugreek seeds exhibit potential as a protective medicinal herb for complementary therapy in cancer patients undergoing chemotherapeutic interventions. This is attributed to the observed protective effect of seed extract, which modifies the cell death induced by cyclophosphamide and the lipid peroxidation mediated by free radicals in the urinary bladder of mice (Laila et al., 2022). The research findings indicate that protodioscin, a chemical derived from fenugreek seeds, can inhibit the growth of HL60 cells by the induction of apoptotic alterations. There has been a proposition that the ingestion of these seeds leads to an increased rate of apoptosis, which may potentially exhibit an impact on breast cancer. The in vitro efficacy of fenugreek seed extract in inducing cytotoxicity has been demonstrated against a diverse range of human cancer cell lines, encompassing neuroblastoma, IMR‐32, and HT‐29 cancer cell lines (Srinivasa & Naidu, 2021).

Diosgenin, a crystalline saponin, naturally occurs in fenugreek seeds. This substance serves as an initial component in the production of steroid hormones, including cortisone and progesterone. According to a report, there exist data suggesting that diosgenin possesses the ability to impede the proliferation of malignant cells in cases of bone cancer. By effectively suppressing the activity of tumor necrosis factor (TNF), it effectively hampers the development and proliferation of bone cells, hence mitigating the potential danger of developing bone cancer (Zhu, Arsovska, & Kozovska, 2020; Zhu, Chen, & Li, 2020).

The study demonstrated that both the crude extract of fenugreek seed powder and its bioactive ingredient diosgenin could inhibit the development of aberrant crypt foci (ACF), which are considered precancerous lesions. The discovery occurred after the examination of the impact of fenugreek seed powder, in conjunction with its bioactive ingredient diosgenin. After the completion of the in vivo experiment, the investigation into the anticancer properties of diosgenin was carried out using a variety of in vitro assays. The examination of HT‐29 human colon cancer cells revealed that diosgenin had cytotoxic effects, leading to cell death, as well as inhibiting cellular proliferation. One approach to substantiate the impact on apoptosis is demonstrated by the examination of apoptotic proteins. Diosgenin was found to induce an upregulation in the expression of caspase‐3, an anti‐apoptotic protein, while concurrently inhibiting the production of the pro‐apoptotic protein BCL‐2 (Jin et al., 2020).

### 3.6 Cardioprotective potential

Numerous studies have demonstrated the potential of fenugreek seeds in reducing cholesterol levels, thereby suggesting their potential efficacy as a cardioprotective agent. The components of fenugreek, namely saponins, diosgenin, galactomannan, coumarin, nicotinic acid, sapogenins, scopoletin, and trigonelline, have been found to exhibit pharmacological effects. Another study involved the administration of isoproterenol to rats to induce myocardial infarction, followed by the evaluation of the effects of fenugreek. According to a study, it was observed that fenugreek exhibited a cardioprotective impact in rats. This effect was evidenced by an augmentation in superoxide dismutase (SOD), catalase (CAT), glutathione peroxidase (GPx), and reduced glutathione (GSH) levels (Almatroodi et al., 2021). Fenugreek exhibits resilient regulatory effects on blood lipid levels, thereby demonstrating efficacy in combating atherosclerosis through the reduction of cholesterol, triglycerides, and LDL levels, as well as the elevation of HDL levels.

Fenugreek, a potent antioxidant, has been shown to provide protection to myocardial tissue against OS and enhance metabolic abnormalities. A study was conducted to investigate the potential protective effect of the hydroalcoholic extract derived from fenugreek seeds in rats with streptozotocin‐induced diabetes. The findings of the study indicate that the administration of fenugreek seed extract exhibits promise in the treatment of diabetic cardiomyopathy through its ability to modulate the expression of genes implicated in the process of apoptosis. According to the report, the downregulation of the pro‐apoptotic *Bax* gene and intercellular adhesion molecule, along with the upregulation of the anti‐apoptotic gene BCL‐2, suggests that fenugreek possesses antioxidant and anti‐apoptotic properties (Bafadam et al., 2021).

The potential cardioprotective effects of fenugreek seeds, which are high in dietary fiber, were assessed in a study involving rats with experimentally induced myocardial infarction. The administration of fenugreek in the diet before therapy exhibited notable advantages, notably in the presence of hypercholesterolemia, due to its impact on the lipid composition of tissues. The findings of the study revealed that the presence of hypercholesterolemia enhanced the extent of myocardial injury during the induction of myocardial infarction. The administration of fenugreek in the diet resulted in the improvement of pathological alterations in cardiac tissue and lipid irregularities in both blood and cardiac samples (Almatroodi et al., 2021).

## 4 FOOD APPLICATIONS OF FENUGREEK SEEDS AND THEIR CONSTITUENTS

Fenugreek is considered among the unique food ingredients as it contains higher amounts of dietary fiber contents. Fenugreek gum is employed in various food products across diverse industries, functioning both as a distinctive component for product stabilization and as a fiber source. The fenugreek protein, especially lysine, is considered as the best substitute for soy proteins (Kumari et al., 2021) and galactomannan is considered the most important soluble dietary fiber of fenugreek (Dhull, Sandhu, Punia, et al., 2020). The utilization of fenugreek seeds in different products has been discussed in Table 2.

**TABLE 2:** Utilization of fenugreek seeds in different products.

Food products	Application strategies	Key findings	References
Buffalo milk yogurts	Fenugreek seed and *Moringa oleifera* flours	Improved yogurt culture feasibility, total polyphenolic content (TPC), antioxidant activity (AOA), nutritional value, functional properties, mineral and antibacterial effect	Dhawi et al. (2020)
Wheat flour rusk	Fenugreek powder	Improved nutritional attributes, acceptability rates, quality, loaf weight, stiffness, antioxidant activity (AOA), fiber, mineral, phytochemical attributes, developed dark color, reduced sensory characteristics and loaf volume	Dhull, Punia, Sandhu, et al. (2020)
Wheat flour and biscuit	Fenugreek powder (10%)	Increased chemical and sensory attributes and hindered degenerative illnesses	El‐Naggar (2019)
Meat products	2% and 4% of fenugreek powder	Increased quality, chewiness, stiffness, gumminess, fat released, and reduced fluid salvation	Frangopoulos (2022)
Injera	Fenugreek flour	Improved nutritional value, enhanced mineral, crude protein, fat, and fiber contents, and reduced microbial growth rate	Godebo et al. (2019)
Beef burger	Seed flour (3%, 6%, 9%, and 12%)	Enhanced sensory attributes and acceptance rate, increased essential amino acid, physicochemical and microbiological quality	Hegazy and Hegazy (2011)
Beef patties	Extract	Antioxidant capacity amended oxidative stability, decreased thiobarbituric acid value, and delayed oxidative rancidity of patties	Hettiarachchy et al. (1996)
Tunisian beef sausage	Fenugreek seed constituents (polar polysaccharide)	Improved storage stability, delayed myoglobin, and lipid oxidation	Ktari et al. (2017)
Upma (semolina‐based)	Fenugreek seed powder, chickpea in different forms	Increased nutritional value, acceptability rate, moisture, fat, protein, fiber, Ca, Fe, decreased gastrointestinal (GI) bleeding, carbohydrates, and calories	Kumari (2013)
Gluten‐free fresh pasta	Chickpea powder, tiger nut, and fenugreek	Improved nutritional value, increased soluble and insoluble fibers, protein %, sensory attributes, developed reddish color and good texture, reduced carbs enzymatic digestion, and glycemic response	Llavata et al. (2020)
Chicken meat patty	Fenugreek powder	Enhanced sensory properties	Qureshi et al. (2018)
Wheat flour	Fenugreek powder (5%, 10%, 15%, and 20%)	Enhanced consistency, viscosities, bulk density, water‐holding capacity, emulsion potentiality, functional properties, produced yellowish color, and reduced melting enthalpy	Sakhare and Prabhasankar (2017)
Cereal‐based products	Fenugreek polysaccharide flour (15%)	Improved physical and sensory attributes and reduced GI properties	Shirani and Ganesharanee (2009)

### 4.1 Diverse uses of galactomannan

Galactomannan has multiple applications due to its unique physicochemical characteristics. It is used as a stabilizer and stiffener in emulsions and its nontoxic attributes have enhanced its usage in food industries, cosmetics, biomedical, pharmaceutical, and textile industries (Vendruscolo et al., 2009). The major applications of galactomannan in food industries include its utilization in curd, frozen and tinned meat products, soups, sauces, seasoning, baby milk formula's formulation, tea whiteners, dietary fiber products, bakery products, various powdered products, fruit gels, and different types of dairy products. This wide application range of galactomannan reveals various functional properties, such as higher viscosity solutions, gel‐forming mixtures, and frozen product stabilization (Cerqueira et al., 2011). Moreover, fenugreek seeds acting as a major source of galactomannan are used as emulsifying, stabilizing, and thickening agents in food industries (Kontogiorgos, 2019).

### 4.2 Bakery products

Fenugreek gum, when added to different types of baked items, can decelerate the staling process in certain meals, including gluten‐free rice cake, by diminishing retrogradation enthalpy and weight loss. Similarly, it regulates the retrogradation of starch, decelerating the process of staling in flatbread whether stored at room temperature or in the refrigerator (Huang et al., 2016).

Punia et al. (2019) demonstrated that the flour of fenugreek seeds is a good source of white flour fortification used for bakery products, such as baked corn‐chips, fried snacks, noodles, tortillas, flatbread, muffins, bagels, bread, cake, and pizza. Different bakery muffins, cakes, pizza, and bread were developed by soluble dietary fiber fortification (8%–10%) on the flour and it improved their attributes. The utilization of this fortified flour to prepare the fried snack in oil reduced the 8%–15% of oil absorption making it acceptable for health‐conscious people. The germinated seeds of fenugreek in powdered form were used in the development of biscuits and bread to enhance the acceptability, mineral contents, nutritional quality, and flour quality and provide reduced gluten contents in final products (Im & Maliakel, 2008). The water extract from germinated seeds was used in wheat flour biscuits to increase the chemical and sensory attributes and to hinder degenerative diseases (El‐Naggar, 2019).

Al‐Shammari et al. reported that the addition of fenugreek gums in cakes and biscuits can potentially improve the dough machinability making it soft enough to leave the mold pans easily and fine slicing without breaking crumbs. Moreover, a 1% fenugreek addition in butter prepared for batter produced the required film developing and binding characteristics that reduced the fats' and oils' absorption and penetration into doughnuts (Al‐Shammari et al., 2022).

### 4.3 Processed meat products

Fenugreek gum is suitable for usage in both hot and cold water environments due to its high water retention capacity. Considering this fact, it is feasible to utilize it as a lubricant, stabilizer, and binder in the production of hotdogs, stuffed meat items, and sausages. Hydrocolloids have been found to serve many beneficial functional roles in the production of meat and meat products. These include controlling the separation of liquid and solid components (syneresis) and preventing fat migration during storage (Eghbaljoo et al., 2022).

### 4.4 Beverages

Fenugreek is potentially being utilized in the beverages due to its various innate attributes required for the thickening and viscosity control of the beverages. Fenugreek gum is preferred in beverages as it is resistant to low pH. Furthermore, fenugreek gums are easily soluble in cold water which helps in enhancing the shelf life of the beverages (Aruna et al., 2018). Zhao et al. (2021) explored the impact of fenugreek gum on different rheological characteristics of quinoa juice at 25°, 4°, and −18°C. The mixture of gum juice depicted the pseudoplastic behavior at an elasticity greater than 1 (tan δ > 1). The viscosity of quinoa juice was also improved with the addition of 0.8% fenugreek gum.

### 4.5 Dairy and dairy products

The fenugreek seed and *Moringa oleifera* seed were utilized in the development of buffalo yogurt to increase the yogurt culture viability, nutritional value, functional properties, minerals, antimicrobial effect, and total polyphenolic content (TPC) (Dhawi et al., 2020). Fenugreek gum is frequently used to stabilize frozen food products such as ice cream due to its high water‐binding capacity. The use of fenugreek gum in cheese products is allowed at a maximum concentration of 3% by weight. This gum serves to increase the quantity of curd solids and facilitate the softening of separated whey. Low‐fat cheese can be manufactured using fenugreek gum at a weight‐to‐volume ratio of 0.0025%–0.01% without any alterations in texture, flavor, or rheology, as opposed to full‐fat cheese (Singh et al., 2018).

### 4.6 Other applications

Gadkari et al. (2018) manufactured gum dispersions by using different sources, i.e., locust beans, guar, and fenugreek seeds, and presented the results that the gums developed by fenugreek had greater gelling attributes in comparison to the guar gums and locust bean gums at the concentration of 1% that can be utilized in various foods production.

Shirani and Ganesharanee (2009) studied the impact of fenugreek powder and its polysaccharide addition on sensory and physical quality attributes and glycemic index of rice‐chickpea‐based extruded snack products. The fenugreek bitter polysaccharides were not acceptable at more than 2% addition due to their bitter taste in extruded products. The fenugreek polysaccharide addition increased the longitudinal expansion and reduced the radial expansion. It increased the water absorption index (WAI) and decreased the water solubility index in comparison to control. The sensory scores indicated that all products containing up to 15% fenugreek polysaccharides were acceptable, as there was no difference in texture, flavor, color, or overall quality of these products.

Wani and Kumar (2018) revealed that fenugreek seeds can be used as hydrocolloids due to their gum and provide encapsulating, gelling, stabilizing, emulsifying, thickening, appealing, and texture‐improving attributes. Therefore, dietary fiber in fenugreek seeds has prominent potential for nutritional improvement in various beverages, yogurts, cereal bars, and dairy products. Dhull, Punia, Kidwai, et al. (2020) reported that fenugreek is used in a variety of sweets, candies, soups, dressings, and milkshakes. It is also utilized in formulations of various capsules and medicines with the combination of various vitamins. Moreover, its total dietary fibers and soluble fibers can be utilized in various fruit juices, spices, and seasonings.

Fenugreek oil exhibits possible antimicrobial properties against a range of microorganisms responsible for foodborne illnesses, hence offering a viable alternative to chemical preservatives. In their study, Dhull et al. (2021) utilized cross‐linked pearl millet starch and fenugreek oil as primary constituents in the development of active starch‐based edible films. The study aimed to mitigate the limitations linked to indigenous starch and substitute artificial preservatives with organic alternatives. The introduction of fenugreek oil into the films led to a significant reduction in inhibition area, with a recorded value of 40.22% for films without cross‐linking and 41.53% for films with cross‐linking, as observed in the experiment conducted against *Escherichia coli*. This study has demonstrated the efficacy of fenugreek oil as an effective antibacterial agent in the formulation of edible films. In a separate investigation, the antibacterial activity of ethanolic extracts of green tea leaves (GTE) and fenugreek seeds (FSE) was evaluated. The study investigated the impact of combining GTE and FSE with chitosan coating on the preservation of Pacific white shrimp (PWS) during refrigerated storage. The findings revealed that the utilization of GTE or FSE during the refrigerated storage of PWS resulted in a statistically significant reduction in total volatile bases, nitrogen, thiobarbituric acid reactive chemicals, total bacterial count, and pH. The results indicated that the use of chitosan coating in combination with GTE or FSE is recommended for improving the quality of PWS during refrigerated storage (Hatab et al., 2018).

## 5 APPLICATIONS OF FENUGREEK SEEDS IN NANOTECHNOLOGY

Spices and culinary herbs have been used in a variety of different applications since ancient times. Their addition to food not only improves its flavor but also its organoleptic properties. This relationship between herbal spices and human health is essential (Ravindran, 2017). Due to the positive effects on humans, researchers from all over the world are conducting extensive research on the efficacy of spices and herbs in the treatment of a wide range of diseases. The medicinal properties of spices and herbs can be improved by encapsulation in nanoemulsions, which may also boost the spices' and herbs' stability and oral bioavailability (Aboalnaja et al., 2016).

### 5.1 Fenugreek seed extract and nanoparticles

Fenugreek seeds are comprised of chemicals that exhibit properties of reducing agents and stabilizers. Previous studies have explored the potential application of fenugreek seed extracts in the development of nanoparticles. Deshmukh et al. (2019) employed fenugreek seed extract as a multifunctional agent for the reduction, capping, and stabilization processes during the manufacture of silver and iron oxide nanoparticles. The confirmation of the effectiveness of fenugreek seed extract as a reducing and capping agent was achieved by the analysis of Fourier transform infrared (FTIR) spectra. In a different research, the synthesis of zinc oxide nanoparticles (ZnO‐NPs) was conducted utilizing an aqueous extract of fenugreek seeds as both a bio‐reducing agent and a capping agent. This approach eliminated the need for traditional reducing chemicals typically employed as reducing agents in such processes. The FTIR analysis confirmed the active involvement of bioactive phytochemical ingredients. Additionally, it has been determined that proteins present in fenugreek aqueous seed extract play a significant part in the bio‐reduction and capping of ZnO‐NPs (Alshehri & Malik, 2019).

Fragoon et al. (2015) employed fenugreek seed extract as a novel approach for the biogenesis of gold nanoparticles (AuNPs) by reduction reaction between an aqueous solution of chloroauric acid and the fenugreek seed extract. A microwave irradiation technique was devised to manufacture biocompatible gold nanoparticles efficiently and safely in a quick manner. The dimensions and morphology of the nanoparticles exhibited a high degree of susceptibility to variations in the concentration of the extract. The utilization of fenugreek extract in the reduction process has resulted in the creation of environmentally sustainable and ecologically friendly gold nanoparticles, which hold potential for application in the field of medicine.

Alwhibi et al. (2018) conducted the synthesis of silver nanoparticles (AgNPs) using a green synthesis procedure, employing fenugreek seed extract as an assisting agent. The resulting nanoparticles were then evaluated for their antifungal and antibacterial activities. The findings demonstrated notable bactericidal efficacy against *Klebsiella pneumoniae*, *Bacillus subtilis*, and *Staphylococcus aureus*, with *E. coli* exhibiting the lowest inhibition. Both *Fusarium equiseti* and *Alternaria alternata* exhibited the highest level of inhibition among the screened fungi.

### 5.2 Fenugreek seed oil encapsulation

Oil encapsulation has drawn increased attention from researchers, as this technique is simpler to handle since it slows oxidation. The consumption of certain oils with beneficial health properties can be challenging due to their distinct flavor or scent. Therefore, the most optimal course of action may involve employing spray‐drying techniques to encapsulate it and utilizing these capsules as a base for the development of a novel product. Maltodextrin and fenugreek mucilage have been used as wall materials in a study that intends to encapsulate fenugreek seed oil (FGSO) to mask the bitter taste as it has numerous health advantages. FGSO has anti‐inflammatory and antioxidant qualities in addition to controlling chronic diabetes and heart disease. Fenugreek seed oil cannot be used directly as oil due to its bitter flavor. To mask the bitter flavor of the oil and make it edible, FGSO can be encapsulated with maltodextrin and fenugreek. Moreover, encapsulation prolongs the shelf life of FGSO and stops bioactive components from degrading (Munshi & Kumar, 2022).

### 5.3 Fenugreek seed oil nanoemulsions

Recent research has shown that nanoemulsions (NEs) made of essential oils have superior antibacterial action compared to the oil itself. In a research investigation, fenugreek oil was employed to produce an NE to explore prospective alternatives to synthetic antimicrobials. The preparation of NE involved the utilization of a composite of three distinct constituents, water, fenugreek oil, and Tween 80. The NEs exhibited promising antibacterial action against all the microbiological strains (*Escherichia coli*, *Bacillus subtilis*, *Staphylococcus aureus*, and *Pseudomonas aeruginosa*) employed in the study and against *P. aeruginosa*, a bacterium that is recognized for its resistance to ampicillin, as well (Mansuri et al., 2023).

The hydrophobic chemicals' bioavailability can be improved by expanding the oil‐in‐water (O/W) NEs. The NEs can be made using botanical extracts and essential oils as natural diabetes medications. In the recent investigation, fenugreek extract, nettle extract, and cumin essential oil were used to make the O/W NEs. The NEs were found to have adequate stability, low cytotoxicity, and antidiabetic effects. The study's findings offer unmistakable proof that NE is a plant medication with antidiabetic effects. A strong candidate for biomedical applications is the NE (Javadi et al., 2021).

### 5.4 Fenugreek seed‐based edible packaging

Consumer desire for natural, safe, high‐quality food goods and packaging that does not impact the environment has increased recently (Wensing et al., 2020). Edible packaging is a packaging material that is suitable for human consumption, consisting of two main categories: edible coating and edible film. Edible compounds are employed in the development of packaging materials, which serve as a main safeguard for food goods against contaminants, ensuring their consumption without adverse consequences (Ramos et al., 2016). In a study, various ratios of taro starch and fenugreek mucilage were employed to fabricate a total of six distinct edible films. The evaluation of films encompassed an analysis of their optical, textural, morphological, microbiological, color, and thermal properties. The films composed of pure taro starch and pure fenugreek mucilage demonstrated superior outcomes across several characteristics. The findings suggested that these edible films had the potential to serve as a key packaging material for food goods (Mohite & Chandel, 2020). Memiş et al. employed the solution casting method to fabricate nanocomposite films consisting of fenugreek seed gum (FSG) and nanoclays. The researchers incorporated varying quantities of nanoclays into the films. The study unveiled that nanocomposite films composed of FSG demonstrated significant antibacterial characteristics against foodborne pathogens, including *Listeria monocytogenes*, *E. coli*, *S*. *aureus*, and *Bacillus cereus*. These antimicrobial qualities were observed, regardless of the type and concentration of clay used in the films. The nanocomposite films containing FSG and nanoclays, with a reinforcement level of up to 5%, exhibit significant promise for utilization in antimicrobial food packaging applications (Memiş et al., 2017).

## 6 CONCLUSION

Fenugreek seeds possess a diverse range of applications in various domains, including health, nutrition, food, and nanotechnology, thereby establishing themselves as a versatile and multifaceted resource. The remarkable nutritional makeup, characterized by a rich presence of essential bioactive components, forms the basis for their prospective therapeutic applications. Extensive studies have demonstrated that fenugreek seeds possess notable qualities that contribute to their antidiabetic, antioxidant, anti‐obesity, hypocholesterolemic, anticancer, and cardioprotective effects. The seeds have found a significant place in the food industry, enriching various products with their unique constituents. Moreover, the exploration of nanotechnology has revealed promising opportunities, utilizing the unique characteristics of seeds for a variety of groundbreaking applications such as nanoparticle synthesis. The review highlighted the extensive implications associated with fenugreek seeds, encouraging additional investigation and advancement in the realms of health, nutrition, and technological progress.

## AUTHOR CONTRIBUTIONS

**Zargham Faisal:** Conceptualization (equal); writing – original draft (equal); writing – review and editing (equal). **Rushba Irfan:** Validation (equal); writing – original draft (equal); writing – review and editing (equal). **Noor Akram:** Validation (equal); writing – original draft (equal); writing – review and editing (equal). **Hafiz Muhammad Irfan Manzoor:** Software (equal); validation (equal). **Mohib Ali Aabdi:** Validation (equal); visualization (equal). **Muhammad Junaid Anwar:** Validation (equal); writing – review and editing (equal). **Sharjeel Khawar:** Formal analysis (equal); validation (equal). **Asifa Saif:** Formal analysis (equal); validation (equal). **Yasir Abbas Shah:** Formal analysis (equal); validation (equal). **Muhammad Afzaal:** Conceptualization (equal); supervision (equal); writing – review and editing (equal). **Derese Tamiru Desta:** Writing – review and editing (equal).

## CONFLICT OF INTEREST STATEMENT

The authors declare no conflict of interest.

## Data Availability

Data will be provided on request.
